# Supplementary figures and images for: A dual interaction between RSV NS1 and MED25 ACID domain reshapes antiviral responses
Source: PLoS Pathog. 2025 Sep 8;21(9):e1012930. doi: 10.1371/journal.ppat.1012930 (PMC12431651; doi:10.1371/journal.ppat.1012930)

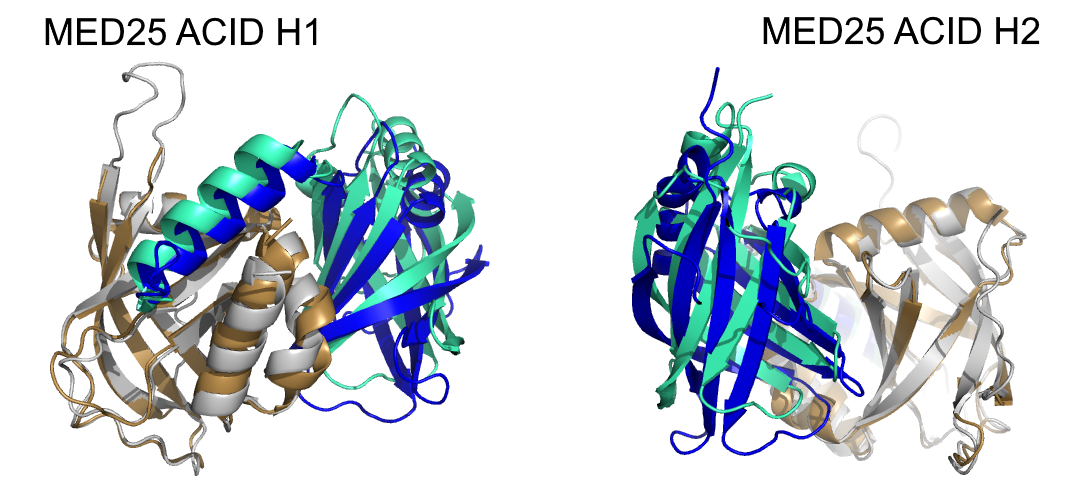

Supplement: S1 Fig — NS1 is in blue, and MED25 ACID in white in the AF model. NS1 is in green cyan, and MED25 ACID in sand in the X-ray crystallographic structure. The RMSD calculated on 774 Cα atoms is 0.787 Å. The two views show the H1 and the H2 interfaces of MED25 ACID. (TIF) [file ppat.1012930.s004.tif]

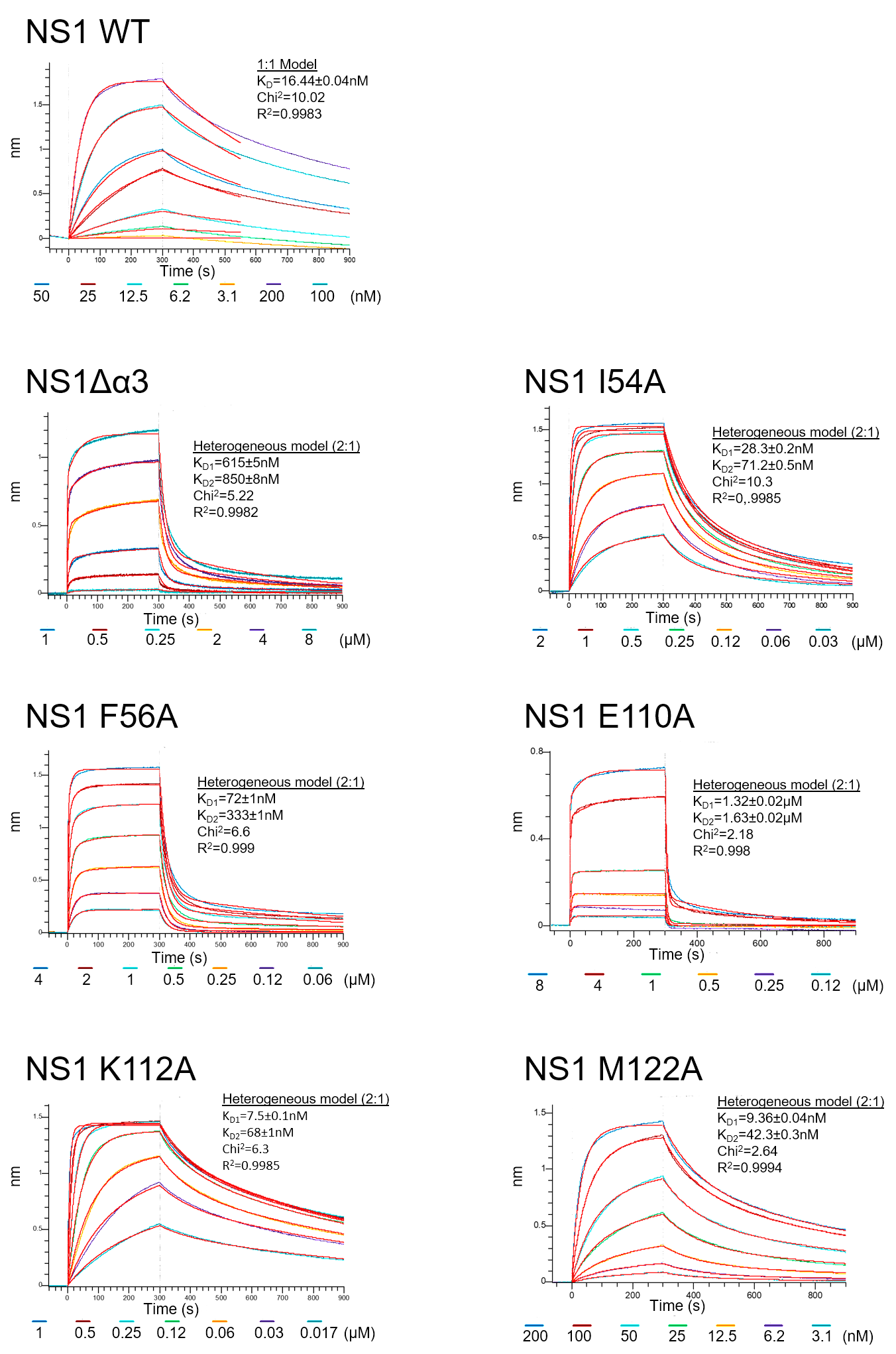

Supplement: S2 Fig — Measurements at 25°C were performed with bound His-tagged MED25 ACID at pH 8.0 and at different concentrations of NS1 proteins, as indicated below each graph. Fitted curves are in red lines, and fitting parameters are indicated in the upper right corner of each graph. The sum of squared deviations Chi2 and the coefficient of determination R2 are indicators for the quality of curve fits. (TIF) [file ppat.1012930.s005.tif]

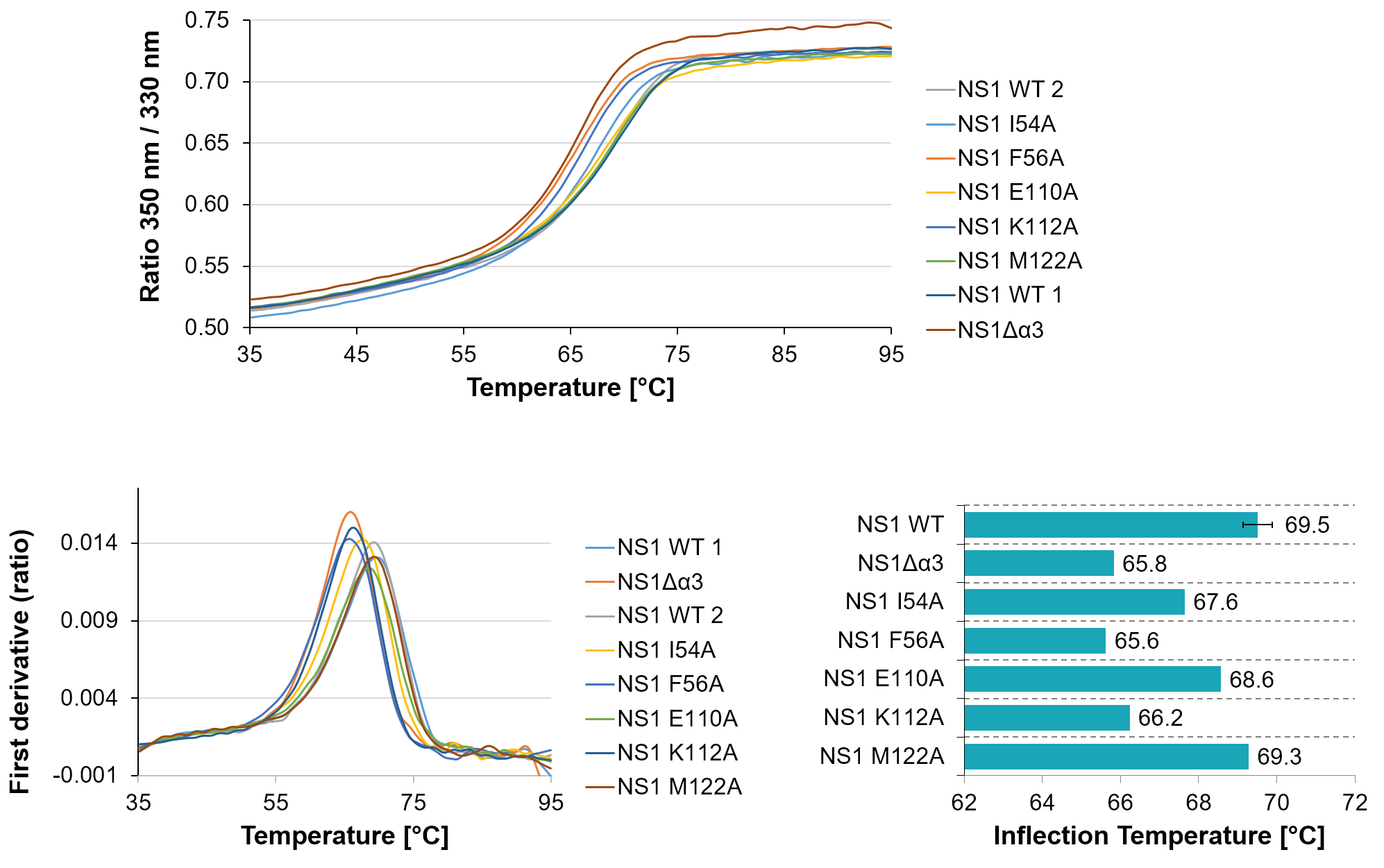

Supplement: S3 Fig — The ratio between fluorescence at 350 and 330 nm and the first derivative are represented as a function of temperature. The inflection temperature is reported in the bar diagram. The value for WT NS1 is a mean value obtained from 2 independent measurements, and the error bar represents the standard deviation. (TIF) [file ppat.1012930.s006.tif]

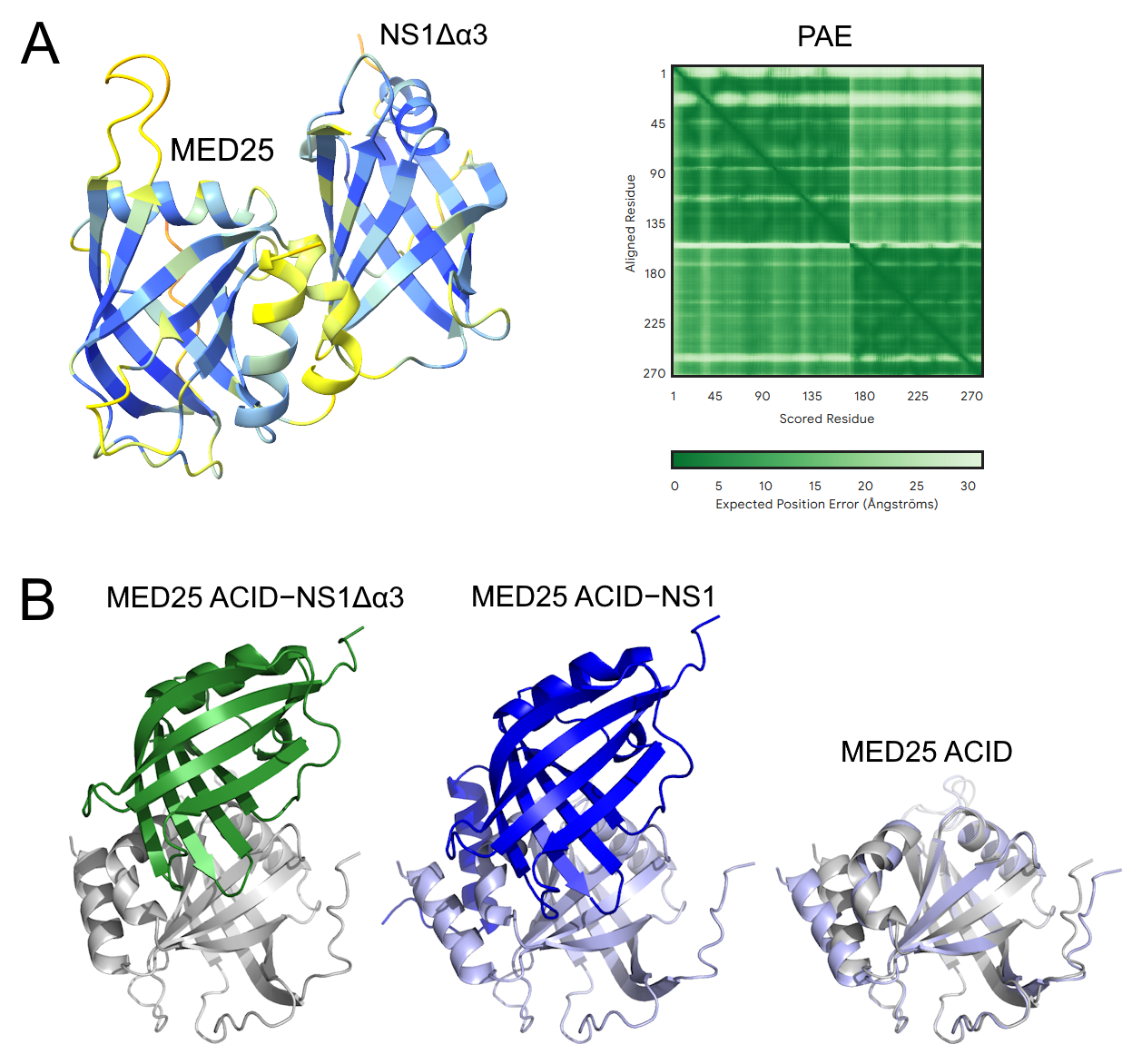

Supplement: S4 Fig — (A) Proteins are in cartoon representation and colored according to the pLDDT (predicted local-distance difference test) confidence score, using the ChimeraX [43] AlphaFold color palette. The predicted aligned error (PAE) matrix for the complex was plotted with a color code from dark green to white representing the expected position error ranging from 0 to 30 Å. (B) The NS1Δα3–MED25 ACID (MED25 ACID in grey and NS1Δα3 in green) and NS1–MED25 ACID (MED25 ACID in light blue and NS1 in blue) complex models were structurally aligned for comparison. (TIF) [file ppat.1012930.s007.tif]

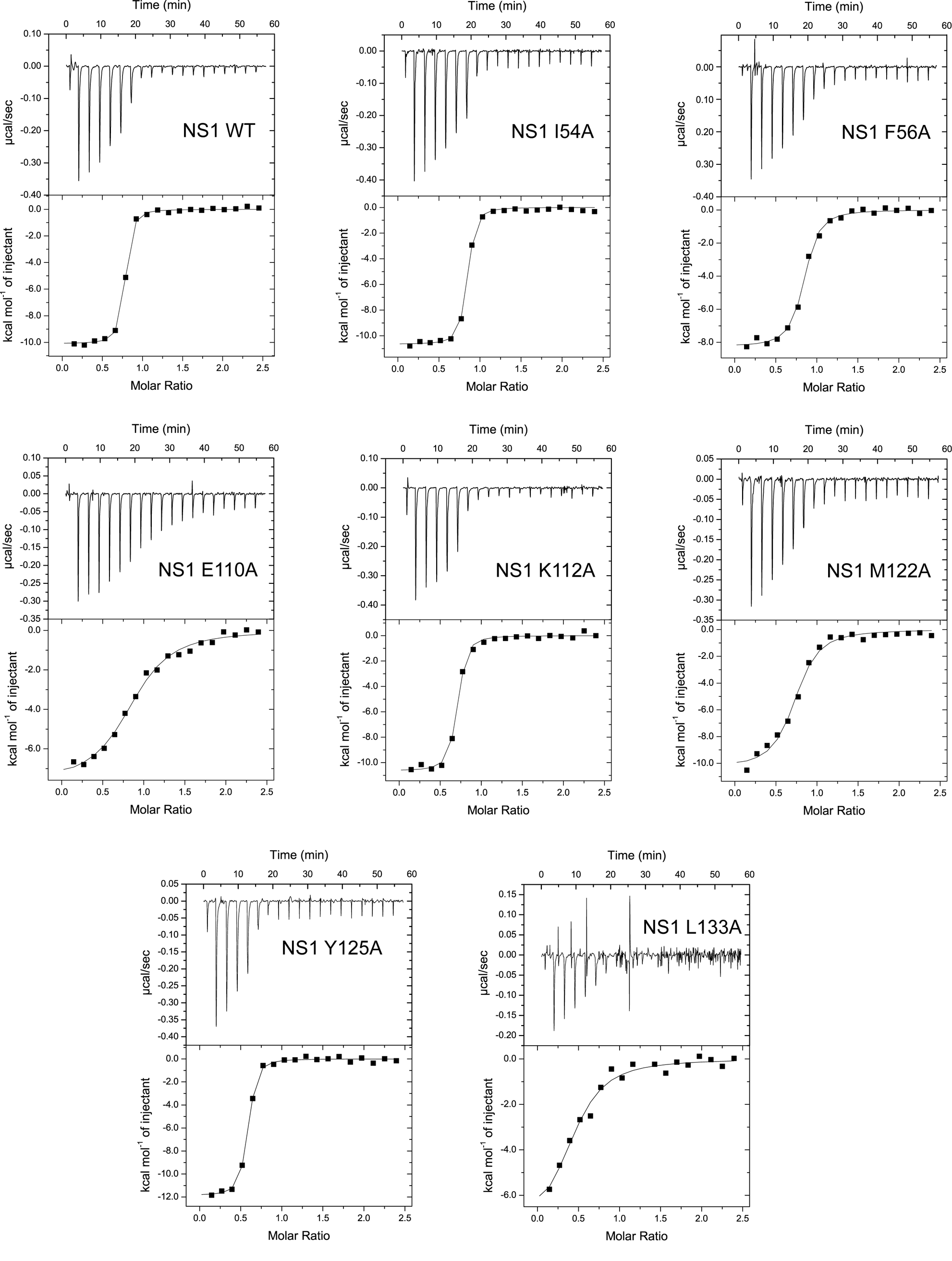

Supplement: S5 Fig — Measurements were carried out in 20 mM Tris pH 8.0, 200 mM NaCl, 1 mM TCEP and at a temperature of 25°C. For each variant, raw binding data are shown on top, and integrated titration curves at the bottom. The NS1 concentration in the calorimeter cell (V = 200 µL) was 20 µM. MED25 ACID at a concentration of 200 µM (for experiments with WT NS1 and NS1Δα3) or 250 µM (for NS1 with single amino acid NS1 mutations) was injected in 2 µL volumes, under stirring at 500 rpm. The duration of injection was 4 s, separated by a delay time of 180 s. (TIF) [file ppat.1012930.s008.tif]

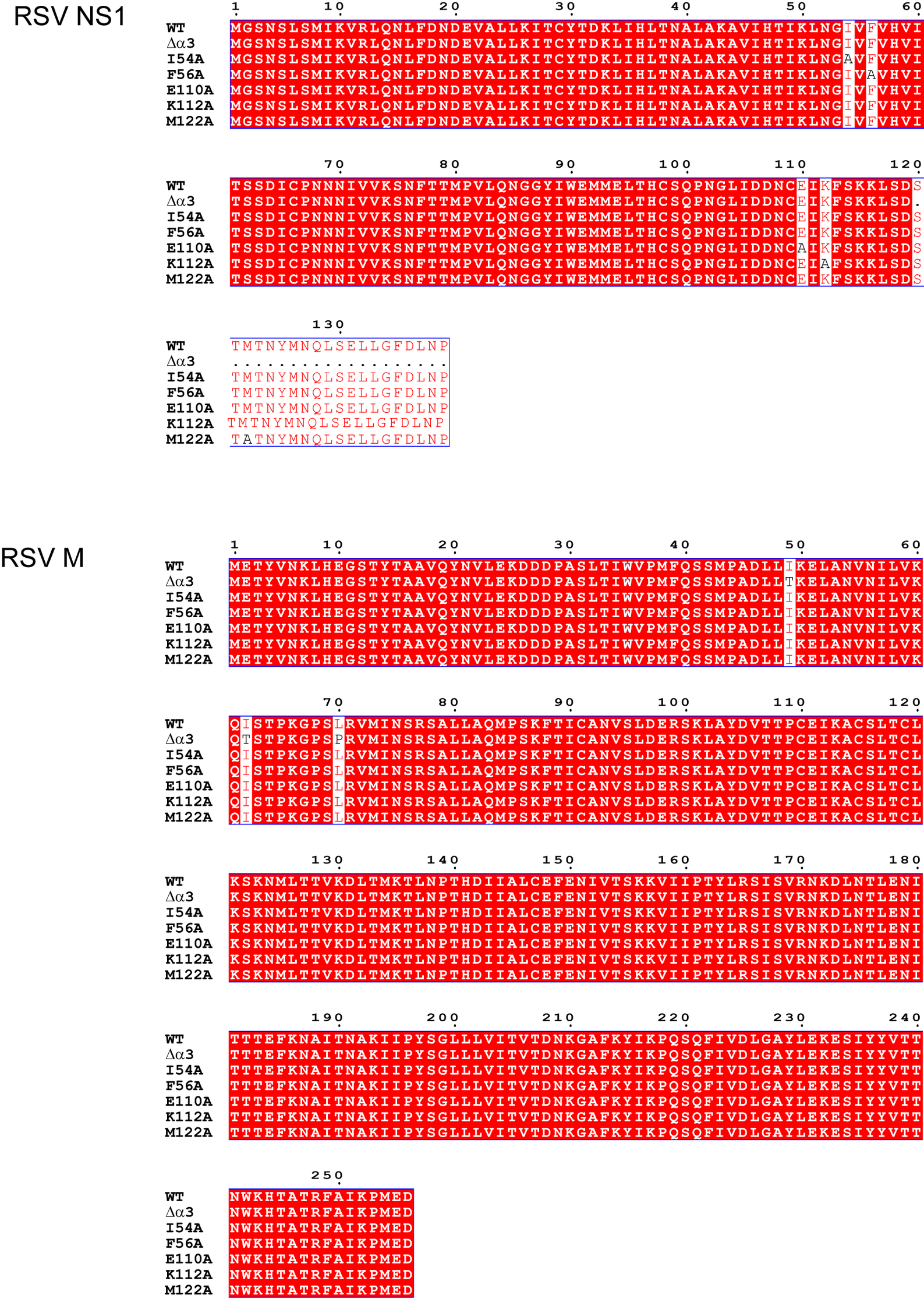

Supplement: S6 Fig — Sequences were obtained from next-generation sequencing of the viral genomes. The alignment was performed with ClustalW and prepared with ESPript3. Residues in red are conserved residues. (TIF) [file ppat.1012930.s009.tif]

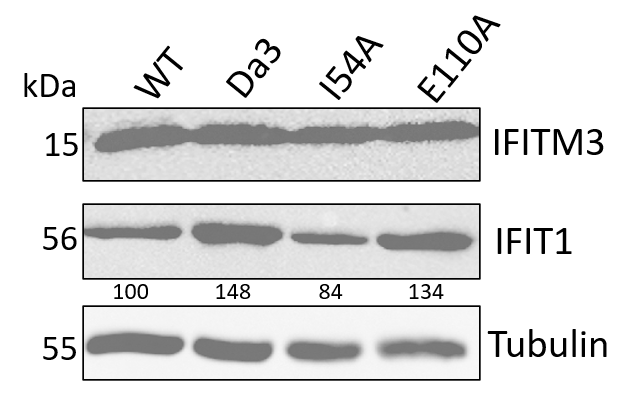

Supplement: S7 Fig — BEAS-2B cells were infected with WT rRSV-mCherry and the most attenuated NS1 mutants at an MOI of 3. At 16 h pi, infected cells were lysed and subjected to a Western Blot analysis using IFITM3, IFIT1, and tubulin antibodies at 1:1000 dilution. IFIT1 protein levels were quantified using ImageJ software. (TIF) [file ppat.1012930.s010.tif]

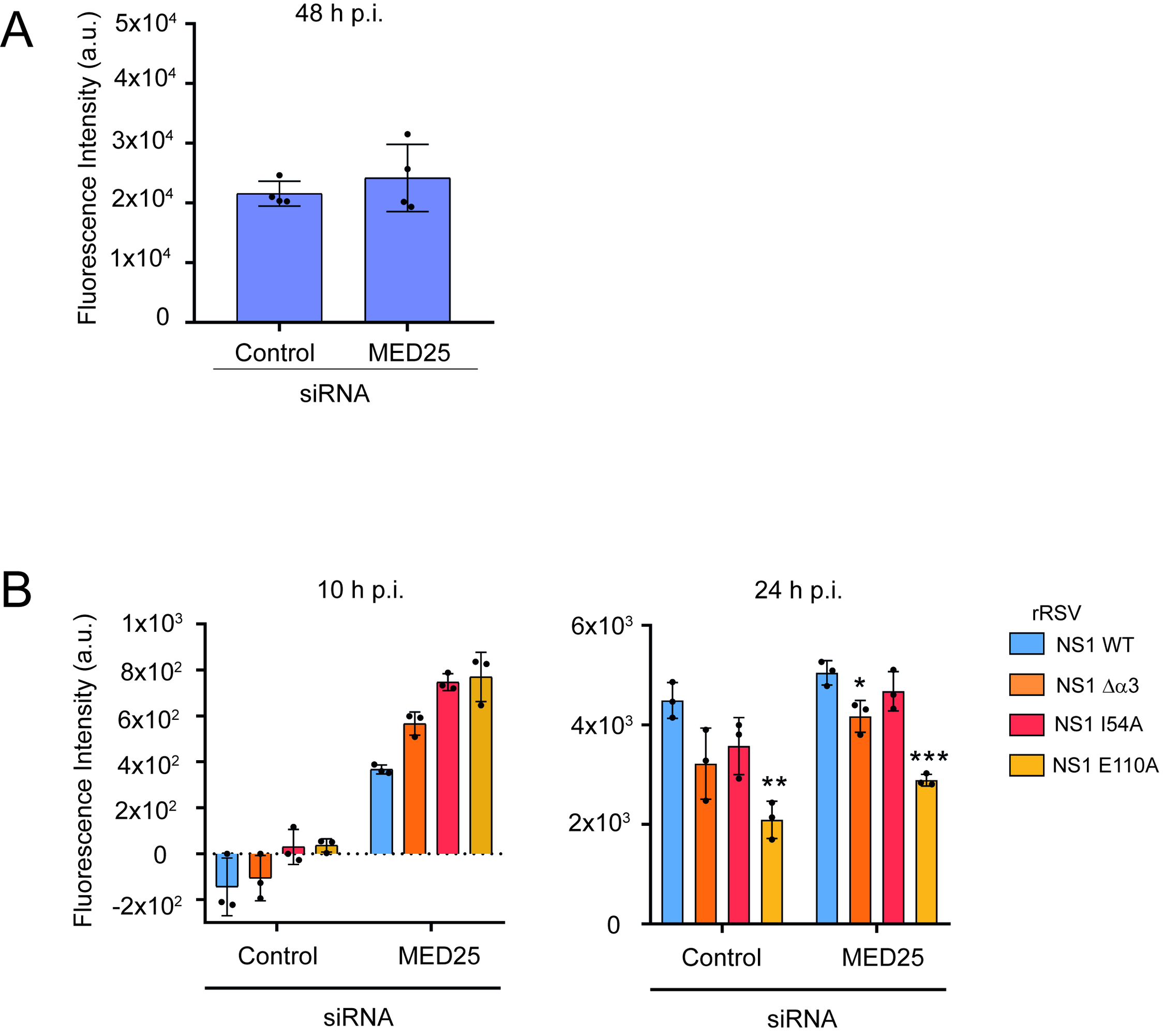

Supplement: S8 Fig — A549 cells were transfected with 10 nM of control siRNA or MED25 siRNA, followed by infection 24 h later with (A) rRSV-mKate2 A2 strain or (B) WT and NS1 mutant rRSV-mCherry Long strain at an MOI of 0.5. RSV replication was quantified (A) at 48 h pi by measurement of mKate2 fluorescence intensity, or (B) at 10 h and 24 h pi by measurement of mCherry fluorescence intensity. Data show means and standard errors of 4 independent experiments in (A) and 3 independent experiments in (B). p < 0.05 *, p < 0.005 **, p < 0.0005 *** (t-test). (TIF) [file ppat.1012930.s011.tif]
