# Supplementary material for: A dual interaction between RSV NS1 and MED25 ACID domain reshapes antiviral responses
Source: PLoS Pathog. 2025 Sep 8;21(9):e1012930. doi: 10.1371/journal.ppat.1012930 (PMC12431651; doi:10.1371/journal.ppat.1012930)
Supplement: Attachment — Submitted filename: Rebuttal letter final.pdf [file ppat.1012930.s013.pdf]

Institut de Chimie des Substances Naturelles
Centre National de la Recherche Scientifique
Université Paris-Saclay

Dr. Christina Sizun
Institut de Chimie des Substances Naturelles
Centre National de la Recherche Scientifique
Avenue de la Terrasse, 91190 Gif-sur-Yvette, France

June 18th, 2025

Dear Editor,

Please, find enclosed our revised manuscript entitled "A dual interaction between RSV NS1 and MED25 ACID domain reshapes antiviral responses".

We are grateful for the opportunity to re-submit our article and thank the two reviewers for their kind consideration, careful reading and constructive comments. We have carried out additional experiments and controls to address several issues raised by the reviewers. Details are provided below in our point-by-point responses in blue. We included these additional data either to the main figures (Fig 1, Fig 5 and Fig 6 contain new information) or to new supplementary figures (Supplementary Fig S1, S5 and S6 in new numbering).

These new data comforted our previous results regarding replication characteristics of our recombinant RSV NS1 protein mutants. They shed new light on the contributions of the RSV NS1 protein subdomains (α/β core domain and C-terminal α3 helix) for interferon induction versus interferon signaling by RSV. Lastly, by investigating more closely the effect of MED25 knockdown, we observed that the effect of MED25 is dependent on the timepoint of viral infection and found evidence for an RSV strain dependence for the effect of cellular MED25.

While we revised our manuscript, a paper from a competing group was published in Nature Communications (Kalita et al, DOI 10.1038/s41467-025-58216-4), underscoring the scope of the interplay between the viral RSV NS1 protein and the human MED25 Mediator subunit. This paper revealed the X-ray structure of the NS1–MED25 complex, which confirmed the high quality of our AlphaFold structural model (comparison shown in Supplementary Fig S1). Kalita et al provide a different perspective on the role of the NS1–MED25 complex, since the authors identified a transcription factor, ATF3, regulated by the NS1–MED25 complex. While Kalita et al focused on the NS1 α3 helix and probed only one residue in the NS1 α/β core domain, i.e. NS1-E110, which we had identified as a major player for the interaction and for downstream functions, we conducted a more extended mutational analysis of the NS1 α/β core domain, and also the mutations for binding to a second NS1 binding partner, TRIM25. We thus provide a more differentiated view of the individual role of the two NS1 subdomains for MED25 binding and modulation of antiviral responses by MED25. In particular extensive mutations of the NS1 α3 helix that disrupt the interaction with MED25, like deletion of α3 or triple mutants used by Kalita et al, might affect NS1 interactions with other cellular partners, and may not point to NS1–MED25-mediated pathways. We suggested a rather simple mechanism for the function of the NS1–MED25 complex, i.e. an inhibition of MED25, based on the high affinity of the interaction with the ACID domain of MED25. We believe that the results obtained by Kalita et al. are compatible with this conclusion, and discuss this in our manuscript.

We are confident that the additional data and the new discussion points have strengthened our manuscript. We hope that it is now meeting the standards for publishing in PLOS Pathogens and thank you in advance for re-considering the manuscript.

Yours sincerely,

Ch. Sizun

Christina Sizun

REVIEWER #1

Reviewer #1 Major Issues:

The MED25 knock down experiments were performed in A549 cells with the RSV-mCherry reporter viruses, as were all other experiments with RSV infection. The authors should also assess the effect of MED25 knock down on the replication of a lab strain of RSV A (or B) or, if available, a clinical isolate of RSV to confirm the observation that RSV replication is restricted in MED25 knock down cells.

We thank the reviewer for this suggestion. Our RSV-mCherry reporter virus was constructed from RSV Long strain. We thus repeated the MED25-knockdown experiments with a second virus, rRSV-mKate2, based on the A2 19F strain, which is the most commonly RSV lab strain used by the community (details are given in Materials and Methods on p36, line 797 and in the paragraph "siRNA transfection and infection" on p 41-42). We now show results for two RSV strains, Long and A2 (**Fig 7** and **Supp Fig S8**). In our hands, the RSV Long strain replicated less in MED25-knockdown A549 cells at a later timepoint of infection, suggesting that MED25 provides an advantage for RSV replication. In contrast, the RSV A2 strain replicated to similar titers in MED25 depleted A549 cells, suggesting that the effect could depend on RSV strain. We comment on this in the Results (p. 26, paragraph starting on line 535) and in the Discussion (p. 32 paragraph starting on line 698).

Reviewer #1 Minor changes:

1. Lines 405-406: HPR > HRP. Done.

2. Lines 454 and 487: "data are from 4 independent experiments". However, for most of the RSV mutants there are 5 or more data points depicted in the graph. Please clarify.
We should have written that the data were obtained for at least 4 independent experiments shown in **Fig 6** and **Fig 7D**. We corrected the figure legends.

3. Line 490: mortality > cell death. Done.

4. Line 662: please specify the source of the A549 cells.
A549 cells were ATCC number CCL-185. We added this information to Materials and Methods (p. 35 line 787).

5. Line 670: please specify in which figure results on infection with RSV A Long are presented.
Our phrasing in Materials and Methods was misleading. We used an rRSV-mCherry virus constructed from RSV Long strain for all experiments (**Fig 5, 6, 7**). New experiments were performed with rRSV-mKate2 from RSV A2 strain (**Supplementary Fig S8**). Details are given in the Materials and Methods (p. 36 line 797 and p.42 line 964). We hope that this is now clearer.

6. Please clarify whether the rescued RSVs were sequence verified.
This was done by next generation sequencing of WT rRSV-mCherry and NS1 mutant genomes, and added to Results (p. 19 line 405). Details are given in Materials and Methods (p. 36 line 802) and in **Supplementary Fig 6**. All NS1 mutations were confirmed. We detected random mutations in the NS1Δα3-rRSV M protein, with three missense mutations (I49T, I62T, and L70P). Two L protein mutations that are normal variants in wildly circulation RSV viruses were found in all rRSV-mCherry viruses with mutated NS1. We discuss the potential impact of the NS1Δα3-rRSV M protein mutations in the Discussion (p. 30 line 658).

7. Line 752: please clarify the statement.
We specified the proteins for which dynamic scanning fluorimetry measurements were done in Materials and Methods (p. 39 line 883).

REVIEWER #2

Reviewer #2 Summary:
This manuscript studies the importance of an interaction between a virus protein, NS1 of Respiratory Syncytial Virus (RSV), that is known to orchestrate IFN antagonism, and a host MED25 protein, which is one of the subunits of the mediator complex. This interaction was known to be key to modulate host transcription in nucleus, a process that is hypothesized to be important for its IFN antagonism. They computationally predict and show that the NS1 C-terminal region interacts with MED25 via the H2 interface of the transcription factor activating TAD domain. They identify key residues in NS1 that are important for this interaction and show that mutating these attenuated RSV replication and induced robust IFN signaling. Despite the thorough biophysical work for predicting and showing protein-protein interaction and identify key NS1 residues for their interaction, the manuscript fails to comprehensively show that these mutations induced higher IFN signaling. Even though the RSV mutants made has a profound replication defect, there is not enough evidence to show the mutants induced IFN signaling as there is no direct measure of increased IFN production. Despite showing increased ISG production via qPCR, the ISGs protein levels should also be quantified to comprehensively show ISG stimulation. Most importantly, a clear link between MED25 and IFN signaling was not established, exacerbated by a replication phenotype in contrast with the previous study of MED25 KO A549 cells. Hence for all these reasons, the manuscript should address the major concerns addressed to be considered for publication.

We thank the reviewer for these valuable comments. We more thoroughly investigated IFN induction and signaling during rRSV-mCherry infection, by measuring mRNA and protein levels of IFN-α,β and λ (**Fig 6A,B**), as well as some ISG protein levels (**Supp Fig 7**) in addition to mRNA levels (**Fig 6C**). These new data show that the NS1 Δα3 rRSV-mCherry mutant induces higher levels of IFN-I and IFN-III, over longer time points, as compared to the single substitution mutants of the NS1 α/β core domain, despite similar disruption of the NS1–MED25 ACID complex. As NS1 has multiple protein partners linked to multiple functions, we cannot exclude that α3 has an additional role as an antagonist of IFN production in the cytoplasm, which is unrelated to its MED25 interaction in the nucleus. We therefore are now more cautious about a correlation between host gene transcription regulation and IFN antagonism. The NS1–MED25 complex may come into play at a later stage of viral infection, when ISGs are transcribed, and could be involved in inflammatory or metabolic responses, which is also one of the conclusions of Kalita et al. Furthermore we also investigated more thoroughly the effect of MED25-knockdown, and were able to draw a link with previously reported data on MED25 KO A549 cells.

Reviewer #2 Major Concerns:
1) It is increasingly becoming more important to show that the virus mutants made via reverse genetics approaches are confirmed for lack of off-target polar mutations both at a plasmid-levels and at a virus level. The observed phenotypes could be dues to random mutations elsewhere in the virus genome and hence validation of these plasmid and virus sequences need to be submitted to ensure that the observed phenotypes were indeed due to the mutations introduced.
This was now done by next generation sequencing of WT rRSV-mCherry and NS1 mutant genomes. We amended Results for it (p. 19 line 405). Details are given in Materials and Methods (p. 36 line 802) and in **Supplementary Fig 6**. All NS1 mutations were confirmed. We detected random mutations in the NS1Δα3-rRSV M protein, with three missense mutations (I49T, I62T, and L70P). The functional relevance of these mutations is presently unknown. Two L protein mutations that are normal variants in wildly circulation RSV viruses were found in all rRSV-mCherry viruses with mutated NS1. We shortly discuss the potential impact of the NS1Δα3-rRSV M protein mutations in the Discussion (p. 30 line 658).

2) Whenever dealing with interaction of proteins at specific domains, it is necessary to include line-diagrams for those proteins showing basic linear domain architecture, so that readers could easily follow. This manuscript will heavily benefit from showing where the intended domains were present in NS1 and MED25 using simple cartoons.

We thank the reviewer for this suggestion and added line diagrams to **Fig 1A** to show the domain organization of MED25 and secondary structures of MED25 ACID and NS1.

3) Whenever showing IFN stimulation, it is necessary to directly quantify IFN levels before showing ISG stimulation. Hence a qPCR showing IFNα/β levels and IFNλ levels (IFNγ are minimally expressed in the cells used and hence it is not necessary to show that) is necessary before showing ISG stimulation.
We thank the reviewer for this comment. We measured mRNA levels of IFN α, β and λ at 10 h and 24 h post infection (**Fig 6A** – p. 24) and comment these data in Results (p. 22, new paragraph starting at line 463). NS1 mutants induced slightly higher IFN levels at 10 h post infection compared to mock, and no difference was seen between WT and NS1 mutants at early time points. It became clear from these new results that the NS1 Δα3 rRSV-mCherry virus was different from the single amino acid substitution mutants, as the induction of IFN was significantly higher and remained high over longer time points. This raises the question whether the NS1 Δα3 deletion is fully relevant to probe NS1–MED25-mediated functions.

4) Along with transcript quantification, it is also important to show protein levels of IFNs and ISGs, as eventually protein level differences are what lead to replication defects and other phenotypes. Hence ELISA or a western blot of the IFNs and ISGs are also important to make these claims.
In addition to mRNA levels, we measured protein concentrations by Luminex for IFN β and λ proteins (**Fig 6B**). The results corroborated the RT-qPCR data. Only NS1 Δα3 rRSV-mCherry induced again higher IFN levels (Results p.22 line 473). We also performed Western blotting for four ISGs (IFIT1, IFITM3, ISG15, and OAS1A) to quantify protein levels, but we were only able to obtain results for IFIT1 and IFITM3 for WT rRSV-mCherry as well as the three NS1 Δα3, I54A, and E110A mutants (**Supp Fig S7**, and new paragraph starting at line 475 in Results). Whereas there was no difference for the four viruses for IFITM3, in agreement with the RT-qPCR results, the result was more contrasted for IFIT1. IFIT1 protein levels were higher for the NS1 Δα3 mutant, in agreement with RT-qPCR. Increased levels of at least this ISG was in line with the fact that this mutant was attenuated. However, the results for NS1 I54A and E110A were less conclusive. We commented these results in the Discussion (p. 31, paragraph starting at line 679).

5) It is confusing to see a decrease in WT virus in MED25KO cells and less of a defect in corresponding NS1 mutants. This could be a virus replication issue and could be caused due to temporal changes. Hence, it is important to show replication of WT and the corresponding RSV mutants at least in 3 different time points (early, mid and late replication timepoint) in A549 cells, as it may help in seeing better differences in replications of the mutants compared to WT.
We agree with the reviewer that this discrepancy needed further investigation. We complemented the data obtained at 48 h pi on MED25-knockdown A549 cells, where we observed decreased replication of WT and mutant rRSV-mCherry in MED25-knockdown cells (**Fig 7C**), by performing measurements at two earlier timepoints: 10 h and 24 h pi (**Supp Fig S8**). At 10 h pi, replication was still too low for the control and the knockdown cells to draw any conclusion. At 24 h pi, there was no significant difference for WT rRSV-mCherry (strain Long) virus between control and MED25-knockdown cells. This indicates that there is indeed a temporal dependence, but also confirms that viral replication is not enhanced in MED25-knockdown cells with our rRSV-mCherry virus. We then measured replication of another virus, i.e. rRSV mKate2 (strain A2) at 48 h pi. In this case we observed replication at the same level in control and MED25 knockdown cells (**Supp Fig S8**). This is in line with the data reported by Van Royen et al. and obtained with RSV A2 (J Virol 2022, DOI 10.1128/jvi.01297-22): the replication difference was much less pronounced than in their RSV B1 strain. We interpret this as evidence for a strain dependence of the effect of MED25 on viral replication. Details are provided in Materials and Methods on p36, line 797 and in the paragraph "siRNA transfection and infection" on p 41-42). We comment on this in the Results (p. 26, paragraph starting on line 535) and in the Discussion (p. 32 paragraph starting on line 698).

Reviewer #2 Minor changes:
1) Line 37 – “IFIT1”. Done.

2) Line 93 – please replace the word “transduce” with another word as transduce has a different meaning in virology. Done.

3) Line 102 – A possible link. Done.

4) Line 338-339 – In this context, Δα3 is used as a negative control as there is a profound replication defect expected. But this is debatable and hence is left to the authors discretion.
Since the wording was misleading, we removed the term “negative control” in this context.

5) Line 405-406 - HRP instead of HPR. Done.
